# Persistent Symptoms (Lasting Longer than 1 Year) in Children Hospitalized with Acute COVID-19 Versus Other Conditions

**DOI:** 10.3390/children11121444

**Published:** 2024-11-27

**Authors:** Marta Conde, Irati Gastesi, Lucía de Pablo, Sara Villanueva-Medina, David Aguilera-Alonso, Ana Esteban, Cristina Epalza, María López, Sara Domínguez-Rodríguez, Pablo Gómez, Álvaro Ballesteros, Carlota Pinto, Marisa Navarro, Carlo Giaquinto, Cinta Moraleda, Alfredo Tagarro

**Affiliations:** 1 Fundación para la Investigación Biomédica e Innovación Hospital Universitario Infanta Sofía y del Henares (FIIB HUIS HHEN), Hospital Universitario Infanta Sofía, San Sebastián de los Reyes, 28702 Madrid, Spain; lpablo@salud.madrid.org (L.d.P.); aeromero@salud.madrid.org (A.E.); mlluengo@salud.madrid.org (M.L.); pgroca@salud.madrid.org (P.G.); alfredo.tagarro@salud.madrid.org (A.T.); 2 Instituto de Investigación 12 de Octubre (imas12), Fundación para la Investigación Hospital 12 de Octubre, 28041 Madrid, Spain; igastesi.imas12@h12o.es (I.G.); sara.villanueva@salud.madrid.org (S.V.-M.); david.aguilera@salud.madrid.org (D.A.-A.); cristina.epalza@salud.madrid.org (C.E.); sara.dominguez@universidadeuropea.es (S.D.-R.); aballesteros.imas12@h12o.es (Á.B.); mariacinta.moraleda@salud.madrid.org (C.M.); 3 Sección Enfermedades Infecciosas, Servicio de Pediatría, Hospital Universitario 12 de Octubre, 28041 Madrid, Spain; 4 Hospital Universitario Gregorio Marañón, 28007 Madrid, Spain; marialuisa.navarro@salud.madrid.org; 5 Centro de Investigación Biomedica en Red de Enfermedades Infecciosas (CIBERINFEC ISCII), 28029 Madrid, Spain; 6 Translational Research Network in Paediatric Infectious Diseases (RITIP), 28009 Madrid, Spain; 7 Pediatric Infectious Diseases, Instituto de Investigación Sanitaria Gregorio Marañón (lisGM), 28009 Madrid, Spain; 8 Department of Medicine, Faculty of Medicine, Health and Sports, Universidad Europea de Madrid, 28670 Madrid, Spain; carlota.pinto@salud.madrid.org; 9 Fondazione Penta ETS, 35127 Padua, Italy; carlo.giaquinto@unipd.it

**Keywords:** post-acute COVID-19 syndrome, children, COVID-19, SARS-CoV-2, chronic fatigue disorder

## Abstract

**Background:** We evaluated the prevalence and characteristics of persistent signs and/or symptoms in children and young people (CYP) one year after hospitalization for acute COVID-19 compared with a control group of CYP hospitalized for other conditions. **Methods:** We conducted an observational study in three hospitals in Madrid, which included a group of children aged between 1 month and 18 years who were hospitalized due to acute COVID-19 from March 2020 to December 2021. We also selected a comparison group of patients hospitalized for other, unrelated conditions within the same month. Eligible participants had no history of COVID-19 at recruitment or during follow-up. Data were collected from clinical records and a standardized questionnaire completed by the patients’ families. The primary outcome was the presence of persistent symptoms one year after hospitalization. **Results:** A total of 96 patients were enrolled and analyzed (50 acute COVID-19 patients and 46 non-COVID-19 participants). Of these, 34/96 (35%) met the criteria for persistent symptoms (CYP: 17/50 (34%) COVID-19 participants and 17/46 (37%) non-COVID-19 participants (*p* = 0.767)). Symptoms persisted ≥12 months in 14/50 (28%) COVID-19 participants and in 7/46 (15%) non-COVID-19 participants (*p* = 0.140). Both before and after admission, all of the participants provided similar ratings for all of the specific items related to emotional welfare, social relationships, and current activities. Readmissions occurred in 11/50 (22%) COVID-19 participants and in 6/46 (13%) non-COVID-19 participants (*p* = 0.267). **Conclusions:** We identified a non-significant difference in the prevalence of persistent symptoms 1 year after hospitalization between children and young people (CYP) with acute COVID-19 and those hospitalized for non-COVID-19-related conditions.

## 1. Introduction

Much attention has been focused on the persistent symptoms that are observed after recovery from coronavirus disease (COVID-19), which are collectively referred to as post-acute sequelae of COVID-19 (PASC), long COVID, or post-COVID condition. Studies on PASC in children have high heterogeneity, with different designs (presence or absence of a control group), inclusion criteria (including confirmed vs. unconfirmed SARS-CoV-2 infection), outcomes (evaluations of symptoms via self-reported or clinical assessment), and follow-up times, among other factors. This heterogeneity is reflected in prevalence rates ranging from 1.7% to 70% [1,2,3]. In adults, previous hospitalization has been associated with an increased risk of PASC (odds ratio [OR]: 2.48; 95% CI, 1.97; 3.13) [4]. Our understanding of this condition is hampered by the paucity of case–control studies, particularly in the pediatric population. In this study, we evaluated the prevalence and characteristics of persistent signs and/or symptoms in children and young people (CYP) one year after hospitalization for acute COVID-19 compared with a control group of CYP hospitalized for other conditions and assessed whether COVID-19 and associated interventions, confers any additional risk of persistent symptoms beyond any risk conferred by being hospitalized and the interventions received there.

## 2. Materials and Methods

We conducted an observational study in three hospitals in Madrid, Spain (Hospital Universitario 12 de Octubre (HU12O), Hospital Universitario Gregorio Marañón (HUGM), and Hospital Universitario Infanta Sofía (HUIS)), nested in a prospective, observational cohort––the Epidemiological Study of COVID-19 (EPICO). Our study group included children between 1 month and 18 years of age who were hospitalized for acute COVID-19 from March 2020 to December 2021 and were included in the EPICO cohort. Patients with multisystem inflammatory syndrome were excluded. For comparative purposes, we selected from admission registers a group of patients hospitalized at HUIS and HU12O in the same month as the participants with COVID-19, who were receiving treatment for non-COVID-19-related conditions, including acute medical- and surgery-related illnesses, who tested negative for COVID-19 at admission and were found to have no reported or recorded history of COVID-19 at recruitment or during follow-up. Both groups of patients were matched 1:1 by month of admission, sex, and age group (<5, 5 to 10, and >10 years). Following the match, the patients’ relatives were contacted, and those who agreed to participate were interviewed by the study personnel, who also reviewed the medical records and confirmed the absence of confirmed infection. The reasons for admission for the comparison group are listed in Supplementary Table S1.

This study was composed of two components. The first was a questionnaire adapted from the ISARIC questionnaire (available upon request) [5]. Data were collected from clinical records upon the participants’ initial hospitalization, new diagnoses after hospitalization, time of diagnosis and current situation, and readmission. Families were also contacted by telephone, and a standardized questionnaire was administered from March 2022 to November 2022, at least one year after the admission of each participant. The questionnaire could be answered online by the caregiver or via a telephone interview with research staff. CYP were permitted to participate in the completion of the form at the discretion of the caregiver. The questionnaire included sections on emotional welfare, social relationships, and activities before and after admission, as well as a section related to current and past physical health. All data were collected using REDCap electronic data collection tools [6]. A specific informed consent form was prepared for non-COVID-19 patients; participants with COVID-19 provided consent at the time of enrolment. The study was approved by the relevant Ethics Committee (code 20/101).

In this study, we adhered to the World Health Organization’s definition of persistent symptoms defined as the development or continuation of new symptoms three months after the initial infection, with these symptoms lasting for at least two months without explanation [7]. The primary outcome was the presence of persistent symptoms one year after hospitalization. The secondary outcome was parental perception of mood and behavioral changes in CYP.

We extracted baseline socio-demographic and clinical characteristics to describe the study population. Data are presented for all participants and summarized by group. Continuous variables were tested for normality using the Shapiro–Wilk test and are reported as the mean and standard deviation (SD) when normally distributed and as the median and interquartile range (IQR) if non-normally distributed. Categorical variables are summarized as frequency counts and percentages. The denominator for each percentage was the number of subjects within the population group without excluding missing observations, unless otherwise stated. The chi-squared test and Fisher’s test were used to test for differences between groups, as appropriate. The Student’s *t*-test was used for normally distributed continuous variables, whereas non-parametric tests (Mann–Whitney U test or Kruskal–Wallis) were used for non-normally distributed data.

All of the hypotheses were tested at the 5% significance level, and *p*-values were rounded to three decimal places. In the summary tables, *p*-values less than 0.001 are reported as <0.001, as implemented in the compare Groups R package (version 4.2.2.2) [8].

A linear regression analysis and Pearson correlation test were employed to evaluate the strength and direction of the association between physical symptoms (independent variable) and emotional symptoms (dependent variable) and to quantify the linear dependency between these two variables. A multivariate logistic model was developed to assess the risk factors associated with the development of persistent symptoms. The model included demographic characteristics (sex at birth and age) and comorbidities (neurological conditions, gastrointestinal problems, heart diseases, respiratory diseases, asthma, eczema, food allergies, other endocrine illnesses, renal problems, excessive weight, and obesity), as well as variables for admission time, severe disease, and COVID-19 vaccination. The results were summarized using ORs with 95% confidence intervals. Variables were selected according to the Akaike information criterion (AIC) using the forward selection method.

To avoid the loss of information and statistical power during the association analysis, missing data were imputed using a non-parametric random forest imputation algorithm. To prevent too many assumptions, only variables with less than 20% missing information were considered for imputation. The other variables were treated as complete cases and any missing information was omitted. Missing observations were balanced between the groups.

## 3. Results

A total of 96 patients were enrolled in our study (50 acute COVID-19 patients and 46 non-COVID-19 participants) after 4 patients had been excluded due to incomplete data. Fifty-seven patients (58.3%) were assigned the male sex at birth. There were no observable differences in baseline characteristics between COVID-19 participants and non-COVID-19 participants (Table 1).

Families were interviewed at a median of 1.89 years (IQR: 1.25–2.07) after hospitalization. The definition of persistent symptoms was met in 34/96 (35%) CYP: 17/50 (34%) COVID-19 participants and 17/46 (37%) non-COVID-19 participants (*p* = 0.767). Symptoms persisted ≥ 12 months in 14/50 (28%) COVID-19 participants and in 7/46 (15%) non-COVID-19 participants (*p* = 0.140).

Non-COVID-19 participants were more likely to present with only one persistent symptom (9/46, 20% vs. 1/50, 2% of COVID-19 participants), but the difference decreased ≥ 12 months after hospitalization (2/46, [4%] vs. 1/50, [2%] of COVID-19 participants). We found that 9 out of 50 (18%) COVID-19 participants and 4/46 non-COVID-19 participants (9%) had ≥3 persistent symptoms after ≥12 months (*p* = 0.174).

**Figure 1:**
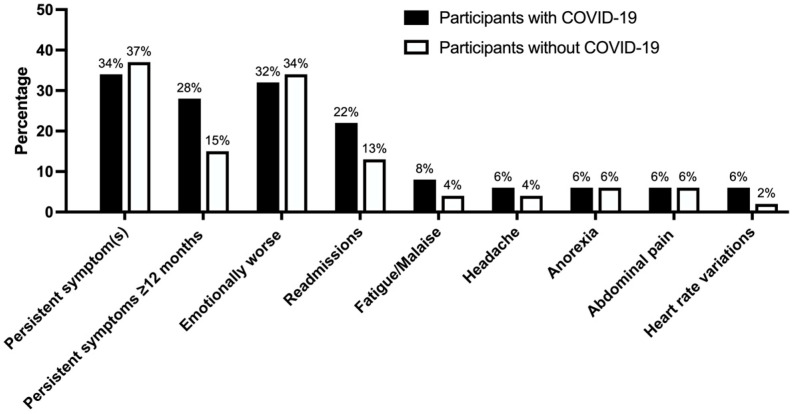
Symptoms 1 year after admission in acute COVID-19 participants and in those with other conditions. None of the comparisons between participants with and without COVID-19 reached statistical significance (*p* < 0.05).

Among the COVID-19 participants, the most common symptoms observed after ≥12 months were fatigue (reported in 4/50 patients (8%)), headache, loss of appetite, abdominal pain, and heart rate variability (reported in 3/50 patients, 6% each). The most common persistent symptoms in participants with no history of COVID-19 were abdominal pain and poor appetite (reported in 3/46 participants, 7% each) (Figure 1).

For emotional and behavioral items, 16/50 (32%) COVID-19 participants reported feeling worse or much worse after admission compared with 16/46 (35%) non-COVID-19 participants (*p* = 0.941). Both groups provided similar ratings before and after admission for all of the specific items related to emotional welfare, social relationships, and current activities (Supplementary Figures S1 and S2).

Among 11/50 (22%) COVID-19 participants, 14 new diagnoses were reported, including neurological (n = 3, 6%), gastrointestinal (n = 3, 6%), pulmonary (n = 2, 4%), and hematological, osteo-muscular, renal, cardiological, allergy-related, and psychiatric conditions (n = 1, 2% each). Among 10/46 (21%) non-COVID-19 participants, there were 10 new diagnoses (1 per participant), comprising gastrointestinal (n = 5, 11%), skin (n = 2, 4%), and osteo-muscular, diabetes, and neurological conditions (n = 1, 2% each). Persistent symptoms 12 months after admission were associated with a new diagnosis (OR 5.16 [95% CI: 1.75; 15.6]). Detailed information on specific diagnoses can be found in Supplementary Table S4.

Readmissions occurred in 11/50 (22%) COVID-19 participants and in 6/46 (13%) non-COVID-19 participants (*p* = 0.267).

A total of 22/50 (44%) participants with COVID-19 were reinfected with COVID-19 during the follow-up. To assess the role of reinfection in persistent symptoms, we compared the prevalence of persistent symptoms and persistent symptoms 1 year after admission in reinfected children (4/22 [18%] and 2/22 [9%]) compared to non-reinfected children (13/28 [46%] and 12/28 [43%]). Children with no history of reinfection were less likely to exhibit persistent symptoms (OR: 0.27, [95% CI 0.06; 0.96]) or to show persistent symptoms after 1 year (OR: 0.15 [95% CI, 0.02; 0.65]). Reinfections occurred 3.2 (SD 4.8) months after admission.

None of the children had been vaccinated against COVID-19 prior to admission, and all of them received the vaccine at some point afterward. The multivariable analysis revealed no association between any of the risk factors analyzed and the development of persistent symptoms. The analyzed risk factors included sex (OR: 0.77; 95% CI [0.07;8.59]), age (OR: 1.23; 95% CI [1.00;1.70]), neurologic conditions (OR: 22.0; 95% CI [0.64;27]), respiratory diseases (OR: 0.00; 95% CI [-;-]), eczema (OR: 0.00; 95% CI [-;-]), and severe disease (OR: 0.00; 95% CI [-;-]).

We identified a weak negative correlation between the number of emotional symptoms and the number of physical symptoms (R = −0.28; β (95% CI) −0.2262 [−0.3844; −0.0680], *p* = 0.005).

## 4. Discussion

The percentage of CYP with persistent symptoms one year after hospitalization was almost twice as high in those hospitalized for acute COVID-19 compared with those hospitalized for other reasons (28% vs. 15%), but the difference did not reach statistical significance (*p* = 0.140). Our findings suggest that persistent symptoms are not unique to COVID-19, but these results should be treated with caution as their interpretation may be complex. This non-significant difference observed in a small sample suggests a certain level of uncertainty.

A growing body of evidence suggests that PASC is associated with the persistence of SARS-CoV-2 RNA and proteins in body tissues and cells, immune activation, or combinations thereof [9,10]. Other viruses, including Epstein–Barr, papillomavirus, measles, enterovirus, herpesvirus, and parvovirus, may remain dormant in humans for prolonged periods, leading to chronic inflammation [11]. This poorly understood interaction between virus and host goes beyond acute infection and may lead to the development of chronic fatigue and several other unspecific and debilitating symptoms [12]. One key factor is the expression of ACE-2 receptors, which are involved in the viral entry of SARS-CoV-2 and are variably expressed in pediatric populations, possibly influencing disease severity and leading to long-term effects. The ACE-2 receptor also plays a crucial role in regulating inflammation by converting angiotensin II (a pro-inflammatory molecule) into angiotensin. During SARS-CoV-2 infection, ACE-2 activity is reduced, facilitating increased inflammation. Autoantibodies against ACE-2 that develop after infection may suppress ACE-2 activity and promote chronic inflammation. This mechanism is a possible contributor of PASC [13]. Additionally, children with underlying comorbidities, such as asthma, obesity, or immunosuppression, may be at higher risk of developing post-COVID conditions due to immune dysregulation [3]. These mechanisms highlight the complexity of post-COVID sequelae in pediatric patients and, thus, warrant further investigation.

On the other hand, persistent, unspecific symptoms such as headache, abdominal pain, or poor appetite may be related to a previous or a new chronic physical or emotional condition that may or may not be associated with the acute episode of hospitalization, as well as being consistent with PASC.

PASC in adults is typically associated with four clinical phenotypes: chronic fatigue syndrome, respiratory syndrome, chronic pain syndrome, and neurosensory syndrome [14]. In our study, the most common persistent symptoms in the COVID-19 patients were fatigue and headache, whereas the patients with unrelated conditions tended to report experiencing abdominal pain and poor appetite, which are not typical of PASC; this result may be explained by the fact that half of the non-COVID-19 participants were admitted for abdominal surgery or because of gastrointestinal concerns. Some participants received new diagnoses during the follow-up. Persistent symptoms were associated with new diagnoses, which, in some COVID-19 participants, were consistent with PASC-associated conditions, such as migraine or anxiety; however, it is unclear if COVID-19 played any role in triggering persistent symptoms.

CYP exhibiting persistent symptoms after hospitalization for non-COVID-19-related conditions have received little attention in the literature. Persistent symptoms may be common in hospitalized children and may vary according to the reason for hospitalization. Further research is needed to ensure that all individuals with post-acute sequelae receive the necessary follow-up care.

The definition of PASC is highly sensitive, but its specificity is low, and there is no gold standard for diagnosis. It is unclear whether our PASC diagnostic instrument was optimal, especially given the subjective nature of the symptoms; more comprehensive scales may prove valuable in identifying differences in symptoms’ intensity [15]. In addition, 35% of the study population were <5 years of age and may have struggled to articulate their symptoms.

We were unable to prove that persistent symptoms are more common in CYP hospitalized for COVID-19 than those hospitalized for other reasons. This finding is consistent with the initial findings of the CLOCK study. In this large national study, the occurrence of any symptoms in adolescents (≥11 years) who tested positive for SARS-CoV-2 was similar to that of their counterparts who tested negative at baseline after three months of follow-up [16]. However, additional findings suggested that ambulatory CYP with COVID-19 had more persistent symptoms than non-COVID-19 participants [17]. In our study, we focused on hospitalized CYP of all ages, whereas most cases of pediatric PASC occur in adolescents after a mild course of disease.

Our study has several limitations. Firstly, it might have been underpowered due to the small sample size; while we acknowledge that the sample was small for a model with so many variables, we believe that excluding key variables might have led to the omission of essential adjustments for known confounders, potentially biasing our results. Thus, we believe that our approach better balanced the risk of omitting important variables against the risk of overfitting. It is also worth noting that the choice of non-COVID-19 participants might have influenced the persistent symptoms reported. Additionally, the inclusion of surgical patients with an over-representation of participants diagnosed with appendicitis, the inclusion of any diagnosis rather than exclusively viral infections, the lack of severity matching, and the likelihood that only control participants with more symptoms agreed to participate limit the study’s results. Some of the non-COVID-19 participants might also have contracted undiagnosed COVID-19 during follow-up, including oligosymptomatic or asymptomatic COVID-19 infection which, though undiagnosed, might have led to the development of PASC that necessitated hospitalization [5,16]. However, the majority of the population has already been infected with COVID-19, and participants with mild suspected disease are rarely tested, which makes conducting studies with larger sample sizes challenging. Our study also presents a possible selection bias, as the group with no recorded history of COVID-19 may not be fully representative of the general population, affecting the generalizability of the results. However, these findings will be useful for new generations of children who are being exposed to COVID-19 for the first time.

The symptoms reported in the COVID-19 group could have lingered due to reinfection, but interestingly, children who had been infected on multiple occasions had lower odds of persistent symptoms. We did not obtain blood samples from participants during follow-up, so we cannot identify any biological correlates such as persistent DNA or proteins associated with symptoms.

## 5. Conclusions

The prevalence of persistent symptoms one year after hospitalization was not significantly different between CYP hospitalized as a result of COVID-19 and non-COVID-19 participants hospitalized for other conditions.

## Figures and Tables

**Table 1:** Characteristics of the participants.

Characteristic	Total	Non-COVID-19 Participants	Acute COVID-19 Participants	*p*-Value
	N = 96	N = 46	N = 50	
Sex				
Male (n, %)	56/96 (58.3%)	27/46 (58.7%)	29/50 (58.0%)	0.847
Age				
Years (Median, IQR)	8.25 [1.98–13.7]	6.53 [0.47–13.0]	8.66 [4.35–14.0]	0.711
<2 years (n, %)	23/89 (25.8%)	13/39 (33.3%)	10/50 (20.0%)	Ref.
2–5 years (n, %)	9/89 (10.1%)	3/39 (7.69%)	6/50 (12.0%)	0.273
6–11 years (n, %)	24/89 (27.0%)	10/39 (25.6%)	14/50 (28.0%)	0.329
12–18 years (n, %)	33/89 (37.1%)	13/39 (33.3%)	20/50 (40.0%)	0.223
Duration from discharge to interview				
Years (Median, IQR)	1.89 [1.25–2.07]	1.57 [1.16–2.08]	1.97 [1.39–2.05]	0.864
Length of hospital admission				
Days (Median, IQR)	4.00 [3.00–6.00]	3.00 [2.00–4.75]	4.00 [3.00–6.00]	0.660
Readmission				
Yes (n, %)	17/96 (17.7%)	6/46 (13.0%)	11/50 (22.0%)	0.267
Participants with new diagnosis after admission				
Yes (n, %)	21/96 (21.9%)	10 (21.7%)	11 (22.0%)	0.927
Number of readmissions				
1 (n, %)	11/17 (64.7%)	4/6 (66.7%)	7/11 (63.6%)	Ref.
2 (n, %)	2/17 (11.8%)	2/6 (33.3%)	0/11 (0.00%)	
≥3 (n, %)	4/17 (23.5%)	0/6 (0.00%)	4/11 (36.4%)	
Severe disease (CPAP, IMV, or PICU)				
Yes (n, %)	3/46 (6.52%)	0/0 (0.0%)	3/46 (6.52%)	
Comorbidities				
Yes (n, %)	44/96 (45.8%)	20/46 (43.5%)	24/50 (48.0%)	0.664
One comorbidity (n, %)	20/96 (20.8%)	10/46 (21.7%)	10/50 (20.0%)	1.000
≥2 comorbidities (n, %)	24/96 (25.0%)	10/46 (21.7%)	14/50 (28.0%)	0.513
Type of comorbidity				
Neurological conditions (n, %)	10/96 (10.4%)	4/46 (8.70%)	6/50 (12.0%)	0.620
Heart diseases (n, %)	5/96 (5.21%)	1/46 (2.17%)	4/50 (8.00%)	0.243
Hematological conditions (n, %)	2/96 (2.08%)	0/46 (0.00%)	2/50 (4.00%)	
Tuberculosis (n, %)	0/96 (0.0%)	0/46 (0.0%)	0/50 (0.0%)	
Respiratory diseases (not asthma) (n, %)	4/67 (5.97%)	2/30 (6.67%)	2/37 (5.41%)	0.841
Food allergy (n, %)	6/96 (6.25%)	4/46 (8.70%)	2/50 (4.00%)	0.384
Allergic rhinitis (n, %)	2/96 (2.08%)	1/46 (2.17%)	1/50 (2.00%)	0.958
Eczema (n, %)	12/96 (12.5%)	5/46 (10.9%)	7/50 (14.0%)	0.661
Asthma (n, %)	7/96 (7.29%)	4/46 (8.70%)	3/50 (6.00%)	0.638
Skin problems (not eczema) (n, %)	0/96 (0.00%)	0/46 (0.00%)	0/50 (0.00%)	
Gastrointestinal problems (n, %)	8/96 (8.33%)	5/46 (10.9%)	3/50 (6.00%)	0.420
Oncological conditions (n, %)	0/96 (0.00%)	0/46 (0.00%)	0/50 (0.00%)	
Immune system diseases (n, %)	3/96 (3.12%)	0/46 (0.0%)	3/50 (6.00%)	
Genetic conditions (n, %)	5/96 (5.21%)	0/46 (0.00%)	5/50 (10.0%)	
Diabetes mellitus (n, %)	1/96 (1.04%)	1/46 (2.17%)	0/50 (0.00%)	
Other endocrine illness (n, %)	3/96 (3.12%)	1/46 (2.17%)	2/50 (4.00%)	0.669
Renal problems (n, %)	2/96 (2.08%)	1/46 (2.17%)	1/50 (2.00%)	0.958
Overweight and obesity (n, %)	5/96 (5.21%)	1/46 (2.17%)	4/50 (8.00%)	0.243
Undernutrition (n, %)	1/96 (1.04%)	1/46 (2.17%)	0/50 (0.00%)	
Rheumatological conditions (n, %)	0/96 (0.00%)	0/46 (0.00%)	0/50 (0.00%)	
Depression (n, %)	0/96 (0.00%)	0/46 (0.00%)	0/50 (0.00%)	
Anxiety (n, %)	1/96 (1.04%)	0/46 (0.00%)	1/50 (2.00%)	
HIV (n, %)	0/96 (0.00%)	0/46 (0.00%)	0/50 (0.00%)	
Persistent symptoms—dichotomized				
Yes (n, %)	34/96 (35.4%)	17/46 (37.0%)	17/50 (34.0%)	0.767
Persistent symptoms				
0 (n, %)	62/96 (64.6%)	29/46 (63.0%)	33/50 (66.0%)	Ref.
1 (n, %)	10/96 (10.4%)	9/46 (19.6%)	1/50 (2.00%)	0.012
2 (n, %)	6/96 (6.25%)	2/46 (4.35%)	4/50 (8.00%)	0.570
≥3 (n, %)	18/96 (18.8%)	6/46 (13.0%)	12/50 (24.0%)	0.329
≥3 persistent symptoms—dichotomized				
Yes (n, %)	18/96 (18.8%)	6/46 (13.0%)	12/50 (24.0%)	0.182
Persistent symptoms ≥12 months—dichotomized				
Yes (n, %)	21/96 (21.9%)	7/46 (15.2%)	14/50 (28.0%)	0.140
Persistent symptoms ≥12 months				
0 (n, %)	75/96 (78.1%)	39/46 (84.8%)	36/50 (72.0%)	Ref.
1 (n, %)	3/96 (3.12%)	2/46 (4.35%)	1/50 (2.00%)	0.679
2 (n, %)	5/96 (5.21%)	1/46 (2.17%)	4/50 (8.00%)	0.207
≥3 (n, %)	13/96 (13.5%)	4/46 (8.70%)	9/50 (18.0%)	0.174
Worse mood and behavior				
Yes (n, %)	32/93 (34.4%)	16/46 (34.8%)	16/47 (34.0%)	0.941
COVID-19 vaccination				
Yes (n, %)	41/96 (42.7%)	21/46 (45.7%)	20/50 (40.0%)	0.584
Before admission (n, %)	0 (0.0%)	0 (0.0%)	0 (0.0%)	.
After admission (n, %)	30/30 (100%)	15/15 (100%)	15/15 (100%)	.

IQR: interquartile range; CPAP: continuous positive airway pressure; IMV: invasive mechanical ventilation; PICU: pediatric intensive care unit admission.

## Data Availability

The original contributions presented in the study are included in the article/Supplementary Materials, further inquiries can be directed to the corresponding author/s.

## Supplementary Materials

The following supporting information can be downloaded at: https://www.mdpi.com/article/10.3390/children11121444/s1, Table S1: Diagnosis of non-COVID-19 participants at discharge; Table S2: Symptoms reported by COVID-19 participants at follow-up and symptom duration in months; Table S3: Symptoms reported by non-COVID-19 participants at follow-up and symptom duration in months; Table S4: Details on new diagnoses received during follow-up; Figure S1: Parental perception of mood and behavior changes in their children after admission (COVID-19 participants). The dark brown color means that parents thought their children were “much worse” compared to before admission, light brown means “worse”, dark green means “much better”, and light green means “better”. Gray means that no change occurred after admission; Figure S2: Parental perception of mood and behavior changes in their children (non-COVID-19 participants). The numbers in the middle part of the bar refer to the proportion of participants who responded with “the same”. The numbers in the left part of the bar refer to the proportion of participants who responded with “much worse” or “worse”. The numbers in the right part of the bar refer to the proportion of participants who responded with “better” or “much better”.
