# Supplementary material for: Persistent Symptoms (Lasting Longer than 1 Year) in Children Hospitalized with Acute COVID-19 Versus Other Conditions
Source: Children (Basel). 2024 Nov 27;11(12):1444. doi: 10.3390/children11121444 (PMC11674934; doi:10.3390/children11121444)

Supplementary Materials

Supplementary Table S1. Diagnosis of non-COVID-19 participants at discharge.

Diagnosis	n	Percentage
Appendicitis	17	37.0
Lower respiratory tract infection or asthma attack	6	13.0
Gastroenteritis	3	6.5
Abdominal pain	3	6.5
Plastic surgery	2	4.3
Seizures	2	4.3
Soft tissue infection	2	4.3
Urinary tract infections	2	4.3
Orthopedic surgery	1	2.1
Allergy to cow milk proteins	1	2.1
Choking	1	2.1
Neonatal conjunctivitis	1	2.1
Prolonged jaundice	1	2.1
Diabetic debut	1	2.1
Fever without a source	1	2.1
Upper respiratory tract infection	1	2.1
Myopericarditis	1	2.1
Total	46	100

Supplementary Table S2. Symptoms reported by the COVID-19 participants at follow-up and symptom duration in months.

Current symptom	Number of patients who reported the symptom	Number of patients with persistent symptom	<1 month	1-2 months	3-4 months	5-6 months	7-8 months	9-11 months	≥12 months	In the last 7 days
Fatigue, n (%)	7/50 (14.00%)	4/7 (57.14%)	2/7 (28.57%)	1/7 (14.29%)	0/7 (0.00%)	0/7 (0.00%)	0/7 (0.00%)	0/7 (0.00%)	4/7 (57.14%)	3/7 (42.86%)
Rhinorrhea, n (%)	4/50 (8.00%)	1/4 (25.00%)	3/4 (75.00%)	0/4 (0.00%)	0/4 (0.00%)	0/4 (0.00%)	0/4 (0.00%)	0/4 (0.00%)	1/4 (25.00%)	1/4 (25.00%)
Disturbance in or total loss of sense of smell, n (%)	4/50 (8.00%)	0/4 (0.00%)	1/4 (25.00%)	1/4 (25.00%)	0/4 (0.00%)	0/4 (0.00%)	0/4 (0.00%)	0/4 (0.00%)	0/4 (0.00%)	0/4 (0.00%)
Headache, n (%)	6/50 (12.00%)	3/6 (50.00%)	3/6 (50.00%)	0/6 (0.00%)	0/6 (0.00%)	0/6 (0.00%)	0/6 (0.00%)	0/6 (0.00%)	3/6 (50.00%)	1/6 (16.67%)
Disturbance in or loss of taste, n (%)	3/50 (6.00%)	0/3 (0.00%)	3/3 (100%)	0/3 (0.00%)	0/3 (0.00%)	0/3 (0.00%)	0/3 (0.00%)	0/3 (0.00%)	0/3 (0.00%)	0/3 (0.00%)
Persistent cough, n (%)	4/50 (8.00%)	3/4 (75.00%)	1/4 (25.00%)	0/4 (0.00%)	1/4 (25.00%)	0/4 (0.00%)	0/4 (0.00%)	0/4 (0.00%)	1/4 (25.00%)	1/4 (25.00%)
Hypersomnia, n (%)	1/50 (2.00%)	1/1 (100%)	0/1 (0.00%)	0/1 (0.00%)	0/1 (0.00%)	0/1 (0.00%)	0/1 (0.00%)	0/1 (0.00%)	1/1 (100%)	1/1 (100%)
Poor appetite, n (%)	4/50 (8.00%)	4/4 (100%)	0/4 (0.00%)	0/4 (0.00%)	1/4 (25.00%)	0/4 (0.00%)	0/4 (0.00%)	0/4 (0.00%)	3/4 (75.00%)	2/4 (50.00%)
Skin rash, n (%)	2/50 (4.00%)	0/2 (0.00%)	2/2 (100%)	0/2 (0.00%)	0/2 (0.00%)	0/2 (0.00%)	0/2 (0.00%)	0/2 (0.00%)	0/2 (0.00%)	1/2 (50.00%)
Diarrhea, n (%)	3/50 (6.00%)	2/3 (66.67%)	1/3 (33.33%)	0/3 (0.00%)	0/3 (0.00%)	0/3 (0.00%)	0/3 (0.00%)	0/3 (0.00%)	2/3 (66.67%)	1/3 (33.33%)
Stomach or Abdominal pain, n (%)	8/50 (16.00%)	5/8 (62.5%)	2/8 (25.00%)	1/8 (12.50%)	2/8 (25.00%)	0/8 (0.00%)	0/8 (0.00%)	0/8 (0.00%)	3/8 (37.5%)	3/8 (37.50%)
Vision problems, n (%)	3/50 (6.00%)	1/3 (33.33%)	0/3 (0.00%)	2/3 (66.67%)	0/3 (0.00%)	0/3 (0.00%)	0/3 (0.00%)	0/3 (0.00%)	1/3 (33.33%)	2/3 (66.67%)
Dizziness, n (%)	0/50 (0.00%)	0/0 (. %)	0/0 (. %)	0/0 (. %)	0/0 (. %)	0/0 (. %)	0/0 (. %)	0/0 (. %)	0/0 (. %)	0/0 (. %)
Joint pain or swelling, n (%)	2/50 (4.00%)	1/2 (50.00%)	1/2 (50.00%)	0/2 (0.00%)	0/2 (0.00%)	0/2 (0.00%)	0/2 (0.00%)	0/2 (0.00%)	1/2 (50.00%)	0/2 (0.00%)
Variations in heart rate, n (%)	3/50 (6.00%)	3/3 (100%)	0/3 (0.00%)	0/3 (0.00%)	0/3 (0.00%)	0/3 (0.00%)	0/3 (0.00%)	0/3 (0.00%)	3/3 (100%)	2/3 (66.67%)
Constipation, n (%)	1/50 (2.00%)	1/1 (100%)	0/1 (0.00%)	0/1 (0.00%)	1/1 (100%)	0/1 (0.00%)	0/1 (0.00%)	0/1 (0.00%)	0/1 (0.00%)	0/1 (0.00%)
Difficulty breathing, n (%)	6/50 (12.00%)	2/6 (33.33%)	3/6 (50.00%)	1/6 (16.67%)	0/6 (0.00%)	0/6 (0.00%)	0/6 (0.00%)	0/6 (0.00%)	2/6 (33.33%)	0/6 (0.00%)
Palpitations, n (%)	2/50 (4.00%)	2/2 (100%)	0/2 (0.00%)	0/2 (0.00%)	0/2 (0.00%)	0/2 (0.00%)	0/2 (0.00%)	0/2 (0.00%)	2/2 (100%)	2/2 (100%)
Feeling nauseous or persistent vomiting, n (%)	3/50 (6.00%)	2/3 (66.67%)	1/3 (33.33%)	0/3 (0.00%)	1/3 (33.33%)	0/3 (0.00%)	0/3 (0.00%)	0/3 (0.00%)	1/3 (33.33%)	2/3 (66.67%)
Chest pain, n (%)	5/50 (10.00%)	1/5 (20.00%)	3/5 (60.00%)	1/5 (20.00%)	0/5 (0.00%)	0/5 (0.00%)	0/5 (0.00%)	0/5 (0.00%)	1/5 (20.00%)	0/5 (0.00%)

Persistent muscle pain, n (%)	5/50 (10.00%)	2/5 (40.00%)	2/5 (40.00%)	1/5 (20.00%)	0/5 (0.00%)	0/5 (0.00%)	0/5 (0.00%)	0/5 (0.00%)	2/5 (40.00%)	1/5 (20.00%)
Problems with balance, n (%)	1/50 (2.00%)	1/1 (100%)	0/1 (0.00%)	0/1 (0.00%)	0/1 (0.00%)	0/1 (0.00%)	0/1 (0.00%)	0/1 (0.00%)	1/1 (100%)	1/1 (100%)
Confusion, n (%)	1/50 (2.00%)	1/1 (100%)	0/1 (0.00%)	0/1 (0.00%)	0/1 (0.00%)	0/1 (0.00%)	0/1 (0.00%)	0/1 (0.00%)	1/1 (100%)	1/1 (100%)
Fainting or blackouts	0/50 (0.00%)	0/0 (. %)	0/0 (. %)	0/0 (. %)	0/0 (. %)	0/0 (. %)	0/0 (. %)	0/0 (. %)	0/0 (. %)	0/0 (. %)

***Total number of patients: N=50**

Supplementary Table S3. Symptoms reported by the non-COVID-19 participants at follow-up and symptom duration in months.

Current symptom	Number of patients who reported the symptom	Number of patients with persistent symptom	<1 month	1-2 months	3-4 months	5-6 months	7-8 months	9-11 months	≥12 months	In the last 7 days
Fatigue, n (%)	6/46 (13.04%)	4/6 (66.67%)	1/6 (16.67%)	1/6 (16.67%)	1/6 (16.67%)	0/6 (0.00%)	1/6 (16.67%)	0/6 (0.00%)	2/6 (33.33%)	3/6 (50.00%)
Rhinorrhea, n (%)	8/46 (17.39%)	2/8 (25.00%)	5/8 (62.5%)	1/8 (12.5%)	1/8 (12.5%)	0/8 (0.00%)	0/8 (0.00%)	0/7 (0.00%)	1/8 (12.5%)	4/8 (50.00%)
Disturbance in or total loss of sense of smell, n (%)	0/46 (0.00%)	0/0 (. %)	0/0 (. %)	0/0 (. %)	0/0 (. %)	0/0 (. %)	0/0 (. %)	0/0 (. %)	0/0 (. %)	0/0 (. %)
Headache, n (%)	4/46 (8.70%)	2/4 (50.00%)	0/4 (0.00%)	2/4 (50.00%)	0/4 (0.00%)	0/4 (0.00%)	0/4 (0.00%)	0/4 (0.00%)	2/4 (50.00%)	2/4 (50.00%)
Disturbance in or loss of taste, n (%)	0/45 (0.00%)	0/0 (. %)	0/0 (. %)	0/0 (. %)	0/0 (. %)	0/0 (. %)	0/0 (. %)	0/0 (. %)	0/0 (. %)	0/0 (. %)
Persistent cough, n (%)	1/46 (2.17%)	1/1 (100%)	0/1 (0.00%)	0/1 (0.00%)	0/1 (0.00%)	0/1 (0.00%)	0/1 (0.00%)	0/1 (0.00%)	1/1 (100%)	0/1 (0.00%)
Hypersomnia, n (%)	1/46 (2.17%)	1/1 (100%)	0/1 (0.00%)	0/1 (0.00%)	0/1 (0.00%)	0/1 (0.00%)	0/1 (0.00%)	0/1 (0.00%)	1/1 (100%)	1/1 (100%)
Poor appetite, n (%)	5/46 (10.87%)	4/5 (80.00%)	0/5 (0.00%)	2/5 (40.00%)	0/5 (0.00%)	0/5 (0.00%)	0/5 (0.00%)	0/5 (0.00%)	3/5 (60.00%)	3/5 (60.00%)
Skin rash, n (%)	1/46 (2.17%)	1/1 (100%)	0/1 (0.00%)	0/1 (0.00%)	0/1 (0.00%)	0/1 (0.00%)	0/1 (0.00%)	0/1 (0.00%)	1/1 (100%)	1/1 (100%)
Diarrhea, n (%)	2/46 (4.35%)	1/2 (50.00%)	1/2 (50.00%)	0/2 (0.00%)	0/2 (0.00%)	1/2 (50.00%)	0/2 (0.00%)	0/2 (0.00%)	0/2 (0.00%)	0/2 (0.00%)
Stomach or Abdominal pain, n (%)	9/46 (19.57%)	4/9 (44.44%)	4/9 (44.44%)	1/9 (11.11%)	1/9 (11.11%)	0/9 (0.00%)	0/9 (0.00%)	0/9 (0.00%)	3/9 (33.33%)	3/9 (33.33%)
Vision problems, n (%)	1/46 (2.17%)	1/1 (100%)	0/1 (0.00%)	0/1 (0.00%)	0/1 (0.00%)	0/1 (0.00%)	0/1 (0.00%)	0/1 (0.00%)	1/1 (100%)	1/1 (100%)
Dizziness, n (%)	1/46 (2.17%)	0/1 (0.00%)	1/1 (100%)	0/1 (0.00%)	0/1 (0.00%)	0/1 (0.00%)	0/1 (0.00%)	0/1 (0.00%)	0/1 (0.00%)	1/1 (100%)
Joint pain or swelling, n (%)	3/46 (6.52%)	2/3 (66.67%)	1/3 (33.33%)	0/3 (0.00%)	0/3 (0.00%)	0/3 (0.00%)	0/3 (0.00%)	1/3 (33.33%)	1/3 (33.33%)	2/3 (66.67%)
Variations in heart rate, n (%)	1/46 (2.22%)	1/1 (100%)	0/1 (0.00%)	0/1 (0.00%)	0/1 (0.00%)	0/1 (0.00%)	0/1 (0.00%)	0/1 (0.00%)	1/1 (100%)	1/1 (100%)
Constipation, n (%)	0/46 (0.00%)	0/0 (. %)	0/0 (. %)	0/0 (. %)	0/0 (. %)	0/0 (. %)	0/0 (. %)	0/0 (. %)	0/0 (. %)	0/0 (. %)
Difficulty breathing, n (%)	2/46 (4.35%)	1/2 (50.00%)	1/2 (50.00%)	0/2 (0.00%)	0/2 (0.00%)	0/2 (0.00%)	1/2 (50.00%)	0/2 (0.00%)	0/2 (0.00%)	1/2 (50.00%)
Palpitations, n (%)	1/46 (2.17%)	1/1 (2.22%)	0/1 (0.00%)	0/1 (0.00%)	0/1 (0.00%)	0/1 (0.00%)	0/1 (0.00%)	0/1 (0.00%)	1/1 (2.22%)	1/1 (2.22%)
Feeling nauseous or persistent vomiting, n (%)	1/46 (2.17%)	0/1 (0.00%)	1/1 (2.22%)	0/1 (0.00%)	0/1 (0.00%)	0/1 (0.00%)	0/1 (0.00%)	0/1 (0.00%)	0/1 (0.00%)	0/1 (0.00%)
Chest pain, n (%)	3/46 (6.52%)	1/3 (33.33%)	1/3 (33.33%)	2/3 (66.67%)	0/3 (0.00%)	0/3 (0.00%)	0/3 (0.00%)	0/3 (0.00%)	0/3 (0.00%)	1/3 (33.33%)
Persistent muscle pain, n (%)	0/45 (0.00%)	0/0 (. %)	0/0 (. %)	0/0 (. %)	0/0 (. %)	0/0 (. %)	0/0 (. %)	0/0 (. %)	0/0 (. %)	0/0 (. %)
Problems with balance, n (%)	0/45 (0.00%)	0/0 (. %)	0/0 (. %)	0/0 (. %)	0/0 (. %)	0/0 (. %)	0/0 (. %)	0/0 (. %)	0/0 (. %)	0/0 (. %)
Confusion, n (%)	1/46 (2.17%)	0/1 (0.00%)	1/1 (100%)	0/1 (0.00%)	0/1 (0.00%)	0/1 (0.00%)	0/1 (0.00%)	0/1 (0.00%)	0/1 (0.00%)	0/1 (0.00%)
Fainting or blackouts	1/46 (2.17%)	0/1 (0.00%)	1/1 (100%)	0/1 (0.00%)	0/1 (0.00%)	0/1 (0.00%)	0/1 (0.00%)	0/1 (0.00%)	0/1 (0.00%)	1/1 (100%)

***Total number of patients: N=46**

Supplementary Table S4. Details on new diagnoses received during the follow-up.

Condition	COVID-19 (n=11 participants)	No COVID-19 (n=10 participants)
Neurological condition	3	1
Migraine	2	0
Epilepsy	1	1
Gastrointestinal	3	5
Recurrent abdominal pain	1	2
Allergy to cow milk proteins	1	1
Suboclusion	1	1
Crohn’s disease	0	1
Hematological	1	0
Anemia	1	0
Osteo-muscular	1	1
Myalgias	1	0
Arthritis	0	1
Psychiatric	1	0
Anxiety	1	0
Renal issues	1	0
Pyelonephritis	1	0
Endocrinology	0	1
Diabetes	0	1
Cardiologic	1	0
Ablation of accessory pathway.	1	0
Pulmonary issues	2	0
Asthma	2	0
Allergy	1	0
Skin problem	0	2

Abscess	0	1
Atopy	0	1

Supplementary Figure S1. Parental perception of mood and behavior changes in their children after admission (COVID-19 participants). Dark brown color means that the parents think that their children are "much worse" compared to before the admission, light brown means "worse", dark green color mean "much better" and light green means "better". Gray means that no change occurred after the admission.

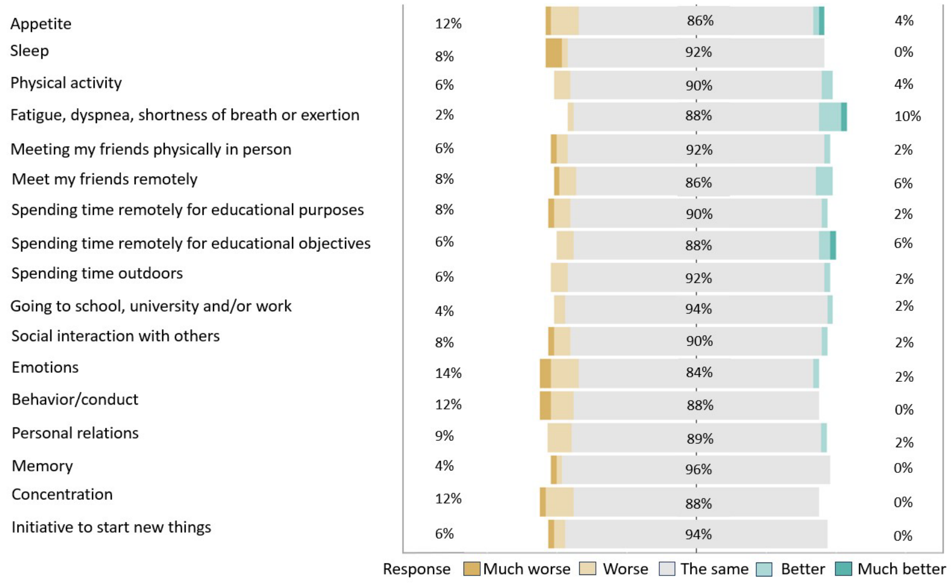

Supplementary Figure S2. Parental perception of mood and behavior changes in their children (non-COVID-19 participants). The numbers in the middle part of the bar refer to the proportion of participants who responded "the same". The numbers in the left part of the bar refer to the proportion of participants who responded "much worse" or "worse". The numbers in the right part of the bar refer to the proportion of participants who responded "better" or "much better".

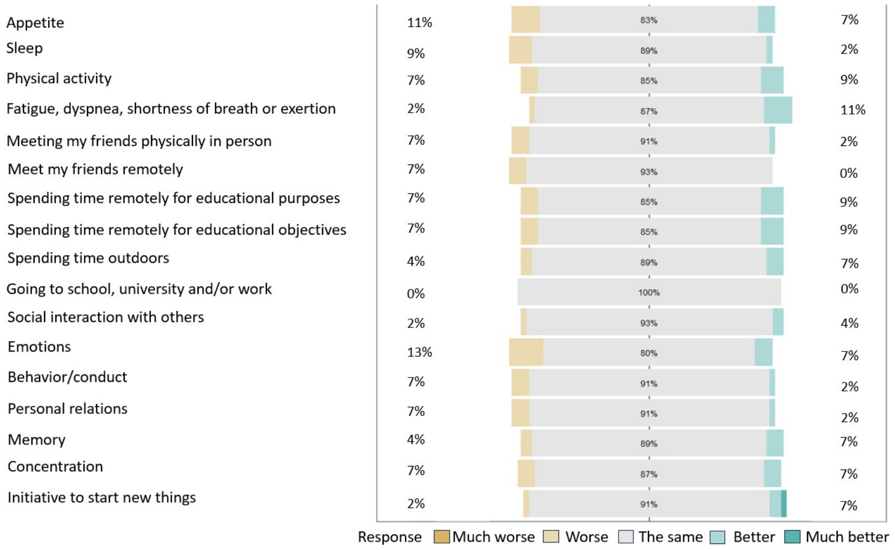

Supplement: Supplementary file 1 [file children-11-01444-s001.zip › children-3268081-supplementary.pdf]
